# Lupus nephritis and U1-RNP-antibodies are associated with low bone mineral density and osteoporosis in patients with systemic lupus erythematosus: baseline findings in a sub-cohort of patients with inflammatory rheumatic diseases

**DOI:** 10.1186/s13075-025-03610-y

**Published:** 2025-07-28

**Authors:** Edgar Wiebe, Elisa Celine Schilling, Dörte Huscher, Andriko Palmowski, Zhivana Boyadzhieva, Sandra Hermann, Burkhard Muche, Mirella Lopez Picazo, Gerhard Krönke, Falk Hiepe, Tobias Alexander, Frank Buttgereit

**Affiliations:** 1 Charité – Universitätsmedizin Berlin, corporate member of Freie Universität Berlin and Humboldt- Universität zu Berlin, Department of Rheumatology and Clinical Immunology, Chariteplatz 1, Berlin, 10117 Germany; 2 Charité – Universitätsmedizin Berlin, corporate member of Freie Universität Berlin and Humboldt- Universität zu Berlin, Institute of Biometry and Clinical Epidemiology and Berlin Institute of Health, Charitéplatz 1, Berlin, 10117 Germany; 3 Section for Biostatistics and Evidence-Based Research the Parker Institute, Bispebjerg and Frederiksberg Hospital, Copenhagen, Denmark; 4 3D-SHAPER Medical, Rambla de Catalunya 53 4-H, 08007 Barcelona Eixample, Spain

**Keywords:** Osteoporosis, Systemic lupus erythematosus, Registries, Fracture risk

## Abstract

**Objectives:**

Patients with systemic lupus erythematosus (SLE) are at higher risk for osteoporosis and fragility fractures. Our study aimed to identify disease-specific factors with impact on bone mineral density (BMD) and the risk of osteoporosis, and to evaluate the effectiveness of DXA-derived 3D femur parameters versus BMD and trabecular bone score (TBS) in discriminating pre-existent fragility fractures.

**Methods:**

We analyzed baseline data of a consecutive subcohort of patients with SLE with current or past GC treatment, fulfilling the EULAR/ACR 2019 SLE classification criteria. We used multivariable linear and logistic regression models to identify BMD- and osteoporosis-related factors. DXA-derived 3D measurements of the femur were performed with 3D-Shaper software. Discriminatory performance of BMD, TBS and 3D femoral parameters for fragility fractures was assessed by AUC values.

**Results:**

Forty-one percent of 110 patients with SLE had osteoporosis. Lupus nephritis (LN) was present in 35% of cases, with 61% (23/38) of these being predominantly classified as classes IV and V. Factors significantly associated with lower BMD included LN classes III and IV, U1-RNP antibodies, higher C-reactive protein, and longer disease duration. Clinical remission, higher Siglec-1 levels, higher body mass index, and higher health assessment questionnaire (HAQ) scores correlated positively with BMD. Osteoporosis was linked to LN, higher age, HAQ, and complement factor 3 levels. Our findings suggest that 3D bone structure analysis may be helpful in discriminating past vertebral fractures.

**Conclusion:**

Disease severity indicated by LN, high CRP, presence of U1-RNP antibodies, and extended disease duration are detrimental to bone health. Moreover, 3D-DXA parameters can be integrated in clinical practise to assess bone health.

**Supplementary Information:**

The online version contains supplementary material available at 10.1186/s13075-025-03610-y.

## Key messages

**WHAT IS ALREADY KNOWN ON THIS TOPIC:**

- SLE patients have lower bone mineral density and higher fracture risk than healthy individuals, but the impact of disease-specific risk factors is unclear.

**WHAT THIS STUDY ADDS:**

- Identifies lupus nephritis (classes III and IV), C-reactive protein, and U1-RNP antibodies to be associated with an increased risk of osteoporosis, while clinical remission appears to benefit bone health.
- Proposes that bone structural parameters as measured by 3D-DXA can be used to discriminate past fragility fractures.

**HOW THIS STUDY MIGHT AFFECT RESEARCH, PRACTICE AND/OR POLICY:**

- Underlines the importance of DXA screening for patients with SLE, especially in those with high disease activity and longer duration of the disease.
- Suggests that new methods for assessing bone structure could help in providing better fracture risk assessments.

**Supplementary Information:**

The online version contains supplementary material available at 10.1186/s13075-025-03610-y.

## Introduction

Systemic lupus erythematosus (SLE) is a chronic autoimmune disease that affects multiple organ systems and is associated with significant morbidity and mortality. Osteoporosis (OP) and fragility fractures (FFx) are significant complications of SLE [1]. Patients with SLE have a threefold higher likelihood of developing OP and fractures compared to the general population [2–5] with a recent meta-analysis estimating an osteoporosis prevalence of approximately 16% [6].

The heightened susceptibility to OP and fractures in patients with SLE is multifactorial, arising from a combination of disease-specific factors, including chronic inflammation and renal involvement, along with traditional contributors such as older age, female sex, low body mass index (BMI), and treatment-related factors like glucocorticoid (GC) use [7]. Therapy with GCs is a well-known risk factor for OP, and recent observational studies estimate that around 30% of patients with SLE receive long-term GC treatment even after 7 years of follow-up [8].

Despite the high prevalence of OP and fractures in patients with SLE, the contribution of disease-specific factors to bone loss in SLE has neither been fully elucidated nor quantified. Furthermore, the gold-standard of Dual-energy X-Ray Absorptiometry (DXA) to measure areal bone mineral density (BMD) may not fully capture the true fracture risk since bone mass does not necessarily amass to bone quality [9, 10], which is determined by structural properties as well [11]. Indeed, many patients with SLE sustain osteoporosis-related fractures, without being diagnosed as “osteoporotic” according to the DXA-based World Health Organisation definition [12], also commonly referred to as the BMD-DXA paradox. Assessment of structural bone properties through (high-resolution) peripheral quantitative computer tomography (HR-pQCT) has been promising in determining fracture risk also in patients with SLE [13], yet is not widely available in clinical practice. More recently, advances in 3 dimensional (3D) modelling have allowed for the measurement of trabecular and cortical parameters from DXA images (19). Moreover, the reconstruction of 2 dimensional (2D) DXA scans to 3D shape and density distribution of the femur, referred to as 3D-DXA, has been shown recently to correlate well with quantitative computer tomography (QCT) for finite element analysis of femoral strength [14].

In this context, we aimed to identify factors associated with BMD and OP including FFx in order to better understand the interplay between disease-specific factors and general risk factors that drive bone loss in this patient population, thereby contributing to a better fracture risk assessment. To achieve this goal, we analyzed the subgroup of patients with SLE enrolled in our prospective monocentric observational cohort study Rh-GIOP (acronym: Glucocorticoid-Induced Osteoporosis in Patients with Chronic Inflammatory Rheumatic Diseases or Psoriasis). Furthermore, we investigated whether measurement of structural bone parameters of the femur has the potential to improve prediction of fracture risk.

## Methods

### Study design and patient involvement

We included all patients with SLE followed in our Rh-GIOP study between 15 July 2015 and 17 January 2022 in the tertiary care university hospital Charité at the Department of Rheumatology and Clinical Immunology, Berlin. For the sub-cohort analysis we present here, only patients fulfilling the American College of Rheumatology/European League Against Rheumatism 2019 SLE classification criteria were included.

Rh-GIOP is a prospective monocentric observational cohort study investigating bone health in consecutive adult patients (≥ 18 years of age) with inflammatory rheumatic diseases and current or prior GC treatment. General Rh-GIOP inclusion criteria are [15]: age ≥ 18 years; diagnosis of a chronic inflammatory rheumatic disease; current or prior GC therapy or indication for upcoming long-term GC treatment; referral to the Charité osteoporosis consultation in line with DVO (Dachverband Osteologie, German umbrella organization for osteology) national osteoporosis guidelines [16]; and ability to provide informed consent. Exclusion criteria involve breastfeeding, pregnant, or lactating patients, as well as patients unable to provide consent.

At study entry, all participants undergo a standardized bone health assessement including a DXA scan and trabecular bone score (TBS) measurement in accordance with national DVO guidelines. Furthermore, various variables pertinent to describing the underlying rheumatic disease and bone health – such as demographic and lifestyle data (e.g. age, sex, BMI, smoking, alcohol, exercise), detailed GC therapy history, disease-specific activity score (including SLE disease activity index, SLEDAI), clinical and technical bone-related parameters, fracture history, fall risk, and relevant laboratory values (e.g. vitamin D, bone turnover markers) are systematically and prospectively collected through patient questionnaires and chart review.

Patients were enrolled at different stages of their SLE disease course; however, the baseline visit following cohort enrollment marked the first standardized osteoporosis assessment, including DXA and TBS.

All necessary ethical and regulatory authorizations were obtained from the local Ethics Committee (Charité-Universitätsmedizin Berlin, EA1/367/14). Rh-GIOP is registered with clinicaltrials.gov (NCT02719314).

A patient representative from the patient organization Deutsche Rheuma-Liga (“German Rheumatism League”) has been involved in the design and management of the Rh-GIOP study and advised the authors in regard to patient-related outcomes.

### Data collection

#### SLE disease activity

Disease activity was assessed using the SLE Disease Activity Index 2000 (SLEDAI-2 K) [17]. Lupus low disease activity state (LLDAS) was defined according to the criteria proposed by Franklyn et al., which include: SLEDAI-2 K ≤ 4, no activity in major organ systems, no new features of lupus activity compared to the previous assessment, a Physician Global Assessment (PGA) ≤ 1 (0–3 scale), and a prednisolone dose ≤ 7.5 mg/day [18].

Clinical remission was defined according to the 2021 DORIS criteria as clinical SLEDAI = 0, PGA < 0.5, and prednisolone ≤ 5 mg/day, with or without ongoing maintenance immunosuppressive therapy [19].

#### Bone densitometry

Areal BMD (aBMD) was measured at the lumbar spine and bilateral proximal femur by DXA. All participants were scanned on a Lunar Prodigy bone densitometer (GE Medical Systems Lunar Corporation, Madison, Wisconsin, USA) per manufacturer recommendations and analysed with the enCORE Software. The results are presented as T − scores. A T-Score ≥ −1.0 was classified as normal, <  − 1.0 to >  − 2.5 as osteopenic (“low bone mass”), and ≤  − 2.5 as osteoporotic. Although Z-scores are typically used to describe BMD in premenopausal women and younger men, osteoporosis was defined according to T-score thresholds in line with World Health Organisation (WHO) criteria and national guidelines (DVO), which are used to guide clinical decision-making in adult populations.

#### Trabecular Bone Score (TBS)

The TBS is a measure of bone texture correlating with the microarchitecture of the lumbar vertebral bodies and is considered an independent predictor of fracture risk [20, 21]. The analysis of the TBS was available from patients enrolled after June 2019 (enCORE V18 iNsight®, Med-Imaps SA, Pessac, France).

#### DXA-derived Three-dimensional (3D) structural parameters of the proximal femur (3D-DXA parameters)

DXA-derived 3D measurements of both femora were obtained using the software 3D-Shaper Research (v2.12, 3D-Shaper Medical, Barcelona, Spain). The software uses a 3D statistical shape and density model of the proximal femur, developed from QCT scans of Caucasian men and women [22]. This 3D model is matched to each patient’s DXA scan to create a subject-specific 3D model of their proximal femur and generate QCT-like parameters [22]. 3D-Shaper software was used to assess trabecular and cortical compartment of the proximal femur individually (18,19). Details of the 3D-Shaper modelling technique can be found elsewhere (18,19). We selected the following variables for our analysis, based on previous publications [23] and clinical relevance: integral (cortical + trabecular) volumetric BMD (integral vBMD; mg/cm^3^), trabecular volumetric BMD (trabecular vBMD; mg/cm^3^), cortical surface BMD (cortical sBMD; mg/cm^2^), cortical thickness (Cth; mm), and cortical volumetric BMD (cortical vBMD; mg/cm^3^). The lowest values in the total hip area from both sides were considered for analysis.

#### Composite outcome measure “Osteoporosis”

In order to reflect the scope of clinical osteoporosis with underlying fractures or patients at risk for FFx despite DXA-values with T-Scores > −2.5, we defined a composite measure of OP by a femoral or lumbar spine T-score of −2.5 or lower and/or history of major osteoporotic fracture (hip, vertebral, humerus or wrist fracture) and/or prescription of anti-osteoporotic drugs in the absence of metastatic bone disease, as previously published [24]. Of note, no patients were prescribed anti-osteoporotic drugs solely as a prophylactic measure for GC treatment. History of fractures was either self-reported and/or verified from patient charts. In case of clinical suspicion of a vertebral fracture, a conventional X-ray examination was performed.

#### Outcomes

Three co-primary outcomes were defined:

1. aBMD, expressed as the lowest T-score measured at the lumbar spine (LS). L1 through L4, total hip (TH) or femoral neck (FN).
2. Osteoporosis (composite outcome measure, see above).
3. Prevalent fragility fractures (any fracture, vertebral fractures, non-vertebral fractures).

#### Statistical analysis

Continuous variables are expressed as mean ± SD, or median and interquartile or interdecile range in case of non-normal distribution. Categorical variables are expressed as frequencies and percentages.

##### Multiple imputation

Multiple Imputation with 10 replications was used to address missing data. Variables categorized a priori as having weak effects on bone mineral density were excluded if values were missing in more than 30% of the patients. These included erythrocyte sedimentation rate (67% missing), parameters of lung function tests (62% missing), echocardiography (68% missing), and duration of biological disease modifying drugs (bDMARD) therapy (85% missing).

To summarize results of backward variable selection, a final model including all variables selected in at least one of the 10 imputed data sets was used without backward selection to produce pooled results; consequently, variables could turn out non-significant in the final tables. For receiver operating characteristics (ROC) analyses, exemplary graphical representation of the results was based on the imputated data set whose area under the curve (AUC) values were closest to the average AUC values of all imputations.

##### Multivariable linear regression

To identify variables associated with the T-score, multivariable linear regression models were performed that included factors preselected according to published evidence and clinical expertise. Our aim was data mining rather than developing a specific prediction model, focusing on identifying variables strongly associated with the respective T-scores from a large pool of potential factors competing within a single model. Collinearity between explanatory variables was neglected. To avoid overfitting, stepwise backward selection was performed to select factors associated with the lowest T-Score. This was done for the minimal T-Score overall, as well as the minimum T-Score for TH and LS, respectively.

##### Multivariable logistic regression

Factors associated with the composite measure “OP” were identified using a logistic regression model. Odds ratios with 95% confidence intervals were reported. As in the linear regression analysis, predefined risk factors according to published evidence were implemented into a multivariable logistic regression model.

##### Discrimination of prevalent fragility fractures

As part of the evaluation of the diagnostic quality of various surrogate markers (T-Score, TBS, and 3D-DXA parameters) for the prediction of fragility fractures, ROC analyses were carried out, and the respective AUC values were determined.

First, AUC values with 95% confidence intervals for T-Score, TBS, and 3D-DXA parameters were calculated and compared. Subsequently, discrimination models were developed using logistic regression analyses that considered the combination of DXA-based T-Scores, TBS, and 3D-DXA parameters for the prediction of three endpoints: any fragility fracture, vertebral fractures, and non-vertebral fractures. Differences between AUC values were evaluated comparing the respective confidence intervals. In addition, tenfold crossvalidation of the logistic regression models including the T-Score, TBS or 3D-DXA parameters and their possible combinations for the three endpoints was performed.

Data analyses were performed using IBM SPSS Statistics version 28.0 (Armonk, NY: IBM Corp)., and R version 4.3.1 (2023–06-16 ucrt).

## Results

### Characteristics of the study population

Data from a total of 110 patients with SLE were analysed. The main characteristics of the study population are presented in Tables 1, 2, 3, 4, and 5. The mean age was 48 (± 14.5 years), and mean BMI was 25.1 (± 5.8). Ninety-two percent of the patients were female, of which 62% were postmenopausal. The majority of patients (86%) received treatment with GCs at baseline visit, mostly in a dose range between 5 and 7.5 mg/d prednisolone equivalent (44%). GC doses above 10 mg per day were observed in 16% of the patients.

**Table 1:** Patients characteristics. **A** Demographics, glucocorticoid therapy and bone status *†

	**SLE (*n* = 110)**		**Missing values n (%)**
**Demographics**			
Age in years, *mean (*± *SD)*	48.1	(± 14.5)	0
Female patients,	101	(92)	0
Past pregnancies, n* (% of total women)*	64	(63)	0
Menopause, n (% of total women)	63	(62)	0
BMI in kg/m^2^, *mean (*± *SD)*	25.1	(± 5.8)	0
**Glucocorticoid therapy**			
Patients with current GC*^1^ therapy, n (%)	94	(86)	0
Current GC dose in mg, *median [IQR]*	5	[2.4–8.5]	0
≤ 2.5 mg/day, n (% of total current GC)	12	(13)	0
> 2.5 and < 5 mg/day	7	(7)	0
≥ 5 and < 7.5 mg/day	41	(44)	0
≥ 7.5 and ≤ 10 mg/day	19	(20)	0
> 10 mg/day	15	(16)	0
Cumulative GC dose in g*^2^, *median [IQR]*	26.8	[13.3–46.6]	0
Duration of GC therapy in years, *median [IQR]*	13.8	[4.9–20.2]	1 (1)
**Bone Status**			
T-score*^3^, *median [IQR]*			
Lumbar spine	−1.2	[−1.9 – 0.1]	2 (2)
Normal*^4^,	52	(48)	
Osteopenia*^5^	42	(39)	
Osteoporosis*^6^	14	(13)	
Femoral neck	−1.1	[−1.8 – −0.5]	1 (1)
Normal	51	(47)	
Osteopenia	51	(47)	
Osteoporosis	7	(6)	
Total hip	−1.1	[−1.8 – −0.2]	1 (1)
Normal	54	(50)	
Osteopenia	46	(42)	
Osteoporosis	9	(8)	
Trabecular Bone Score (TBS)*^7^, *median [IQR]*	1.40	[1.32–1.50]	1 (1)
Normal*^8^,	59	(54)	
Partly degraded *^9^	32	(29)	
Degraded *^10^	18	(17)	
Osteoporotic Fractures*^11^ *no. of patients (%)*	32	(29)	0
Vertebral	11	(10)	
Non-vertebral	28	(26)	

^*^Categorical variables are presented as number and per cent of valid observations (%) unless otherwise noted

^†^Continuous variables are presented as mean values with SD unless otherwise noted

SLE: systemic lupus erythematosus; BMI: body mass index; GC: glucocorticoids; TBS: Trabecular Bone Score

SD: standard deviation; IQR: interquartile range

^*^^1^ GCs include both oral and intravenous application forms of prednis(ol)one, methylprednisolone and modified- release prednisone. All doses are given in prednisone equivalent

^*^^2^ Cumulative GC dose is an estimate calculated from information provided by the patient with the help of patient charts for the entire duration of GC therapy

^*^^3^ BMD and T-Score are measured using the DXA scanner GE Healthcare Lunar Prodigy DF + 15,629

^*^^4^ T-Score ≥ −1 is considered normal

^*^^5^ Osteopenia is defined as a T-Score < −1 and > −2.5

^*^^6^ Osteoporosis is defined as a T-Score ≤ −2.5

^*^^7^ TBS (Trabecular Bone Score) was calculated using the enCORE V18 iNsight® Software

^*^^8^ Normal is defined as a TBS ≥ 1.350

^*^^9^ Partly degraded is defined as a TBS > 1.2 and < 1.350

^*^^10^ Degraded is defined as a TBS ≤ 1.2

^*^^11^ History of fractures was self-reported and/or verified from patient charts, if available. In case of clinical suspicion of a vertebral fracture, a conventional X-ray examination was performed. Fractures were adjudicated under osteoporotic fractures when having occurred due to inadequate trauma or fall from standing height

**Table 2:** Patients Characteristics. **B** Risk factors for osteoporosis *†

	**SLE (*n* = 110)**		**Missing values n (%)**
**Disease activity**			
HAQ, median [IQR]	0.3	[0.0–1.1]	2 (2)
S-CRP mg/l, median [IQR], *ref. [*< *5]*	1.8	[0.6–6.2]	8 (7)
SLEDAI-2 K, median [IQR]	4	[2–8]	2 (2)
Clinical remission	37	(34)	2 (2)
Disease duration (yrs), mean (± SD)	16.3	(± 9.9)	0
Use of care services*^1^	66	(64)	6 (6)
**Age**			
Age group			
< 50 yrs	64	(58)	0
50–64 yrs	31	(28)	0
65–79 yrs	15	(14)	0
> 80 yrs	0	(0)	0
Underweight (BMI < 18.5 kg/m^2^)	11	(10)	0
Underweight DVO guideline (BMI < 20 kg/m^2^),	17	(16)	0
**Family History,**			
For osteoporosis	20	(19)	3 (3)
For osteoporotic fractures	9	(8)	1 (1)
**Co-medication,**			
Proton pump inhibitors	47	(43)	0
NSAIDs	14	(13)	0
Antidepressants	13	(12)	0
Oral antidiabetics	1	(1)	0
Insulin	2	(2)	0
Antihyperuricemic drugs	3	(3)	0
Estrogens (female pts. only)	3	(3)	0
Concomitant diseases*^2^**,**			
Osteoarthritis	11	(10)	0
Diabetes Type 1	0	(0)	0
Diabetes Type 2	4	(4)	0
Dyslipidemia	7	(6)	0
Depression	10	(9)	0
Renal insufficiency	10	(9)	0
Hyperuricaemia/Gout	3	(3)	0
Hyperthyroidism	3	(5)	0
Hyperparathyroidism	8	(9)	17 (16)
Raynaud syndrome	42	(70)	50 (46)
Antiphospholipid syndrome	33	(73)	0

*Categorical variables are presented as number and per cent of valid observations (%) unless otherwise noted.

†Continuous variables are presented as mean values with SD unless otherwise noted.

*1 Use of care services comprises any level of care received, including low-level support. The latter applied for most patients.

*2 Concomitant diseases: shown are diseases or medications that are either particularly common and/or variables considered to have a ‘weakly expected’ impact on the T-score. To avoid overfitting, diseases or medications were not considered in our model when case numbers were low (such as history of transplantation, chronic obstructive pulmonary disease, antiepileptic therapy, heart failure, aromatase inhibitors and hypogonadism).

BMI, body mass index; HAQ, Health Assessment Questionnaire; NSAID, non-steroidal anti-inflammatory drug; S-CRP, serum C reactive protein.

**Table 3:** Patients Characteristics. **C** Protective factors for osteoporosis*

	**SLE (*n* = 110)**		**Missing values n (%)**
**Treatment of underlying disease**			
csDMARDs*^1^	96	(87)	0
Azathioprine (% of total csDMARDS)	33	(30)	0
Cyclophosphamide	7	(6)	0
Mycophenolate mofetil	23	(21)	0
Antimalarial drugs	72	(66)	0
Methotrexate	6	(6)	0
Biologics	20	(18)	0
Rituximab	3	(3)	0
Belimumab	17	(16)	0
tsDMARDs*^2^	3	(3)	0
**Anti-osteoporotic therapy**			
Vitamin D supplementation	103	(94)	0
Calcium supplementation	7	(6)	0
Bisphosphonates*3	10	(9)	0
Alendronate	5	(5)	0
Ibandronate	1	(1)	0
Risedronate	2	(2)	0
Zoledronic acid	2	(2)	0
Denosumab	2	(2)	0
**Behavioral**			
Sun exposure (> 30 min/day)	47	(43)	0
Non-smoker (never)	57	(52)	0
Former smoker	29	(26)	0
Active smoker^*4^	24	(22)	0
No Alcohol consumption	59	(54)	0
Regular physical exercise	93	(85)	0

Categorical variables are presented as number and per cent of valid observations (%) unless otherwise noted.

*1 csDMARDs include azathioprine, chloroquine, ciclosporin, cyclophosphamide, hydroxychloroquine, leflunomide, methotrexate, mycophenolate mofetil and sulfasalazine.

*2 tsDMARDs include tofacitinib, baricitinib and apremilast.

*3 Bisphosphonates include alendronate, ibandronate, risedronate, pamidronic acid and zoledronate.

*4 Active smoking is a known risk factor for OP and is only listed in this table for completeness of information.

csDMARD, conventional synthetic disease-modifying antirheumatic drug; tsDMARD, targeted synthetic disease-modifying antirheumatic drug.

**Table 4:** Patients Characteristics. **D** (part 1) Disease-specific factors

	**SLE (*n* = 110)**		**Missing values n (%)**
Laboratory tests*			
Hemoglobin g/dl, *ref. [12–17]*	12.9	[11.3–13.9]	19 (17)
Leukocytes/nl, *ref. [3.9–10.5]*	6.4	[4.8–8.3]	19 (17)
Thrombocytes/nl, *ref. [150–370]*	263	[205–329]	19 (17)
S-25-hydroxy vitamin D nmol/l, *ref. [50–150]*	87	[69–102]	11 (10)
Vitamin D deficiency*^1^, n (%)	10	[7.0–14.3]	11 (10)
S-Osteocalcin ng/ml, *ref. [11–46.0]*	10.4	[11.1–20]	26 (24)
S-BAP µg/l, *ref. [5.5–38.0]*	15.0	[49.0–80.0]	40 (36)
S-AP U/l, *ref*. [35–130]	58.5	[16.0–33.0]	36 (33)
Gamma-GT U/l, *ref. [5–61]*	24	[25.0–92.0]	16 (15)
Urinary Deoxypyridinoline nmol/l, *ref. [*< *64]*	49	[7.0–14.3]	40 (36)
Serum Creatinine mg/dl, *ref. [0.5–1.2]*	0.8	[0.7–0.9]	5 (5)
Urinary Total Protein mg/24 h, *ref. [*< *150]*	91	[47.4–312.9]	52 (47)
Urinary Albumin mg/d, *ref. [*< *30]*	9	[3.0–115.5]	92 (84)
C3 mg/l, *ref. [900–1800]*	870	[733–1103]	20 (18)
C4 mg/l, *ref*. [100–400]	150	[97.5–220]	20 (18)
ESR mm/h, *ref [3–30]*	22	[6.0–41.5]	74 (67)
ANA titers 1:, *ref*. [≤ 1:80]	640	[320–2560]	3 (3)
Anti-dsDNA antibody U/ml, *ref. [*< *20]*	26.8	[12.9–64.3]	24 (22)
CD 169/Siglec-1-expression on monocytes Ag/cell, *ref. [*< *2400]*	3038.0	[1332–7789]	45 (41)
Positivity for U1-RNP antibodies [negative], n (%)	15	(15)	11 (10)
Positivity for anti-SS-A/Ro antibodies, *ref. [negative]*	39	(40)	12 (11)
Positivity for anti-SS-B/La antibodies, *ref. [negative]*	10	(10)	12 (11)
Rheumatoid factor IgA U/ml, *ref. [*< *20]*	14.4	[3.6–27.7]	23 (21)
Rheumatoid factor IgM U/ml, *ref. [*< *20]*	6.3	[2.0–19.8]	23 (21)
ACPA antibodies U/ml, *ref. [*< *20]*	16.0	[2.0–19.8]	31 (28)

* Results of Laboratory test are reported as median [IQR] unless otherwise noted.

*1 Vitamin D deficiency is defined as serum 25-hydroxy vitamin D level below the lower range of normal <50 nmol/L.

S-BAP, serum bone alkaline phosphatase; S-AP, serum alkaline phosphatase; Gamma-GT, gamma-glutamyltransferase; C3, complement factor 3; C4; complement factor 4; ESR, erythrocyte sedimentation rate ANA, anti-nuclear antibody; ACPA, anti-citrullinated protein antibodies

**Table 5:** Patients Characteristics. **D** (part 2) Disease-specific factors

	**SLE (*n* = 110)**		**Missing values n (%)**
**Organ manifestations**			
**Renal**			
Lupus nephritis, *no. of patients (%)*	38	(35)	0 (0)
Class I: minimal mesangial glomerulonephritis (% of LN-patients)	0	(0)	10 (26)
Class II: mesangial proliferative glomerulonephritis	2	(5)	
Class III: focal glomerulonephritis	3	(8)	
Class IV: diffuse proliferative nephritis	17	(45)	
Class V: membranous glomerulonephritis	6	(16)	
Active Lupus nephritis, *no. of total LN-patients (%)*	21	(55)	8 (21)
**Pulmonary**			
Pleuritis, *no. of patients (%)*	11	(10)	4 (4)
Active Pleuritis, *no. of total patients with pleuritis (%)*	6	(55)	
Past Pleuritis	5	(46)	
Pleural effusion, *no. of patients (%)*	12	(11)	2 (2)
Active Pleural effusion, *no. of total patients with pleural effusion (%)*	7	(58)	
Past Pleural effusion	5	(42)	
Pulmonary function testing, median [IQR]			
Total lung capacity (TLC, in L)	4.9	[4.2–5.6]	68 (62)
Vital capacity (VC, in L)	2.8	[2.4–3.5]	68 (62)
Forced expiratory volume (FEV1, in L)	2.2	[2.0–2.8]	68 (62)
Diffusion Capacity of the Lungs for Carbon Monoxide (DLCO, in mmol/(min*kPa))	1.1	[1.0–1.3]	73 (66)
**Cardiac**			
Pericarditis, *no. of patients (%)*	6	(5)	2 (2)
Active Pericarditis, *no. of total patients with pericarditis (%)*	1	(17)	
Past Pericarditis	5	(83)	
Pericardial effusion	8	(7)	2 (2)
Active pericardial effusion, *no. of total patients with pericardial effusion (%)*	2	(25)	
Past pericardial effusion	6	(75)	
Libman-Sacks-Endocarditis, *no. of patients (%)*	3	(3)	4 (4)
Active Libman-Sacks-Endocarditis, *no. of total patients with endocarditis (%)*	1	(33)	
Past Libman-Sacks-Endocarditis	2	(67)	
Myocarditis, *no. of patients (%)*	1	(1)	3 (3)
Active myocarditis, *no. of total patients with myocarditis (%)*	0	(0)	
Past myocarditis	1	(100)	
Cardiac function parameters, median [IQR]			
Left ventricular ejection fraction (LVEF, in %)	60	[55.0–63.5]	60 (55)
Tricuspid annular plane systolic excursion (TAPSE, in cm)	2.2	[2.0–2.5]	75 (68)
**Neuropsychiatric symptoms**, *no. of patients (%)*	13	(12)	3 (3)
Active neuropsychiatric symptoms, *no. of total patients with Neuro lupus (%)*	2	(15)	
Past neuropsychiatric symptoms	11	(85)	
**Musculoskeletal**			
Arthralgia*, no. of patients (%)*	48	(45)	4 (4)
Arthritis	21	(20)	5 (5)
Myositis	4	(4)	4 (4)
**Hematologic**			
Autoimmune hemolytic anemia	11	(10)	94 (86)
Thrombocytopenia	5	(5)	6 (6)
Leukopenia	7	(7)	6 (6)
**Mucocutaneous**			
Mucosal Ulcers	6	(6)	7 (6)
Non-scarring alopecia	30	(30)	11 (10)
Discoid lupus lesions	21	(20)	7 (6)

Average disease duration was 16.3 (± 9.9) years. Most patients (71%) had lupus low disease activity (LLDAS) [18] and thirty-seven patients were in clinical remission according to DORIS (SLEDAI 0 and prednisolone ≤ 5 mg/day) [25]. The median SLEDAI-2 K was 4 (interquartile range, IQR: 2–8).

Lupus nephritis (LN) was present in 35% of the SLE patients, of whom 55% (21/38) had active nephritic disease at baseline osteoporosis screening visit. Class IV and V accounted for most LN cases (61%, 23/38). Pulmonary, cardiac or neuropsychiatric manifestations were rare (all around 10%).

Serologically, 44% of patients with SLE had positive rheumatoid factor IgA or IgM or positive anti-citrullinated protein antibodies (Table 1-D). Fifteen percent of the patients were positive for U1-RNP-Antibodies. Median Siglec-1 levels on monocytes as a surrogate marker for Type-I interferon activity were within the normal range.

Based on the DXA-derived minimum T-Scores at LS, TH and FN, osteoporotic DXA values according to the WHO definition were observed in 19%, most frequently at the lumbar spine. Using commonly suggested thresholds of TBS, 17% of the patients showed a degraded microarchitecture characterized by a TBS of ≤ 1.2, while in 29% it was partly degraded (TBS > 1.2 and < 1.350).

Low-trauma vertebral fractures were noted in 10% of the patients, while 26% had past non-vertebral fractures (5% had both vertebral and non-vertebral fractures).

According to our composite outcome measure “osteoporosis”, 41% of the cohort was identified has having OP.

Ten percent of the patients had vitamin D deficiency as defined by values of 25-OH-Vitamin D of < 50 nmol/l, although nearly all patients supplemented vitamin D (94%), and 11% of the patients received specific anti-osteoporotic treatment, mostly in the form of bisphosphonates.

### Factors associated with low bone mineral density

Multivariable linear regression analysis on the minimum T-score revealed lupus nephritis class III and IV (reg. coefficient (95%CI): −0.745 (−1.395;−0.095)), the presence of U1-RNP antibodies (−0.750 (−1.314;−0.187)) as well as high C-reactive protein (CRP, −0.015 (−0.026;−0.003)) and longer disease duration (−0.037 (−0.056;−0.018)) to be significantly associated with low aBMD (Table 6).

**Table 6:** Multivariable linear regression for aBMD

	**I) Min. T-Score (any site)**			**II) Min. T-Score (lumbar spine 1–4)**			**III) Min. T-Score (total hip)**		
	Reg. coefficients	(95% CI)	*p-value*	Reg. coefficients	(95% CI)	*p-value*	Reg. coefficients	(95% CI)	*p-value*
BMI	**0.045**	**(0.014; 0.076)**	**0.005**	**0.079**	**(0.035; 0.123)**	**0.001**	**0.070**	**(0.038; 0.101)**	**0.001**
CRP	**−0.015**	**(−0.026; −0.003)**	**0.015**	**−0.016**	**(−0.032; −0.001)**	**0.033**	**−0.017**	**(−0.029; −0.006)**	**0.003**
SLEDAI-2 K	**0.052**	**(0.009; 0.096)**	**0.018**	0.050	(−0.000; 0.099)	0.051	**0.068**	**(0.028; 0.107)**	**0.001**
Anti-osteoporotic therapy	**−0.585**	**(−1.159; −0.011)**	**0.046**	**−0.945**	**(−1.738; −0.153)**	**0.019**	**−0.925**	**(−1.486; −0.363)**	**0.001**
U1-RNP antibodies	**−0.750**	**(−1.314; −0.187)**	**0.009**	−0.301	(−0.987; 0.385)	0.389	**-**	**-**	**-**
Clinical remission	**0.447**	**(0.037; 0.857)**	**0.033**	**-**	**-**	**-**	**0.490**	**(0.089; 0.892)**	**0.017**
Elevated Siglec-1 levels	**0.558**	**(0.150; 0.967)**	**0.007**	**-**	**-**	**-**	0.379	(−0.015; 0.773)	0.059
Disease duration (years)	**−0.037**	**(−0.056; 0.018)**	**0.001**	**-**	**-**	**-**	**-**	**-**	**-**
Class III & IV nephritis	**−0.745**	**(−1.395; −0.095)**	**0.025**	**-**	**-**	**-**	**-**	**-**	**-**
GC-duration (years)	**-**	**-**	**-**	**-**	**-**	**-**	**−0.020**	**(−0.038; −0.003)**	**0.025**
HAQ	**0.307**	**(0.078; 0.536)**	**0.009**	**-**	**-**	**-**	**-**	**-**	**-**
Alkaline phosphatase	**-**	**-**	**-**	**−0.008**	**(0.016; −0.000)**	**0.042**	**-**	**-**	**-**
Osteocalcin	**-**	**-**	**-**	−0.019	(−0.039; 0.001)	0.057	**−0.019**	**(−0.034; −0.004)**	**0.015**
Family history of OP	**-**	**-**	**-**	**-**	-	-	−0.398	(−0.873; 0.077)	0.100
Sun exposure	**-**	**-**	**-**	**-**	-	-	0.310	(−0.042; 0.662)	0.084

Results from the multivariable linear regression models for T-Score at I) any site, II) lumbar spine (L1-L4) and III) total hip. Factors are sorted by descending number and then average strength of effect. Variables with at least one significant impact in multivariable linear regression of the lowest (minimum = min.) are shown. Significant impact factors are highlighted in bold

*BMI* Body Mass Index, *CI* confidence interval, *CRP* C-reactive Protein, *GC* Glucocorticoids, *HAQ* Health Assessment Questionnaire

Conversely, clinical remission (defined as SLEDAI-2 K = 0 and GC dosage ≤ 5 mg prednisone equivalent per day) was positively associated with aBMD (+ 0.447 (0.037;0.857)), as were elevated Siglec-1 levels on monocytes as surrogate for Type-I interferon activity (+ 0.558 (0.150;0.967)), a higher BMI (+ 0.045 (0.014;0.076)), and higher health assessment questionnaire (HAQ, + 0.307 (0.078;0.536)) scores (Table 2).

### Factors associated with osteoporosis

In multivariable logistic regression analysis, active LN (OR (95%CI): 7.42 (1.256;43.868)) was strongly associated with OP in patients with SLE. Additionally, higher age (1.06 (1.02;1.10)), lower HAQ (0.29 (0.12;0.68)) and higher complement factor 3 levels (1.002 (1.000;1.005)) were found to be significantly related to the presence of OP (Table 7).

**Table 7:** Multivariable logistic regression analysis for OP

	**Odds ratio (95%CI)**	***p-value***
**(Active) Lupus nephritis**	**7.424 (1.256; 43.868)**	**0.027**
**HAQ**	**0.286 (0.120; 0.682)**	**0.005**
**Age (years)**	**1.060 (1.020; 1.100)**	**0.003**
**Complement factor 3**	**1.002 (1.000; 1.005)**	**0.048**
Alkaline phosphatase	1.017 (0.995; 1.039)	0.131
ANA titer	1.000 (1.000; 1.000)	0.117
Proteinuria	0.998 (0.996; 1.001)	0.123
BMI	0.919 (0.832; 1.015)	0.094

Results of the multivariable logistic regression. Factors are sorted by descending number and then average strength of association. Significant impact factors are highlighted in bold

*OP* osteoporosis, composite score, *HAQ* Health Assessment Questionnaire, *ANA* anti-nuclear antibodies, *BMI* Body Mass Index

Neither current GC use nor cumulative GC dose were significantly associated with aBMD or clinical OP. GC duration was only associated with low aBMD at the femoral region.

### Discriminatory value of DXA-derived 3D structural parameters of the femur for pre-existent fragility fractures

**Fig. 1:**
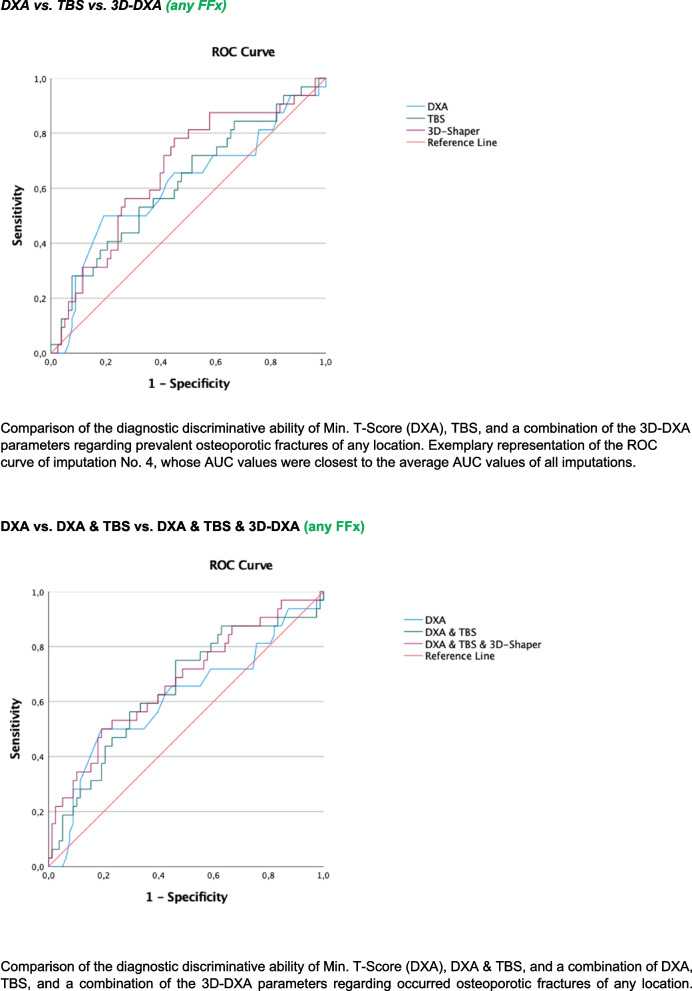
Discrimination performance of DXA, TBS, and 3D-DXA parameters for any fracture

**Fig. 2:**
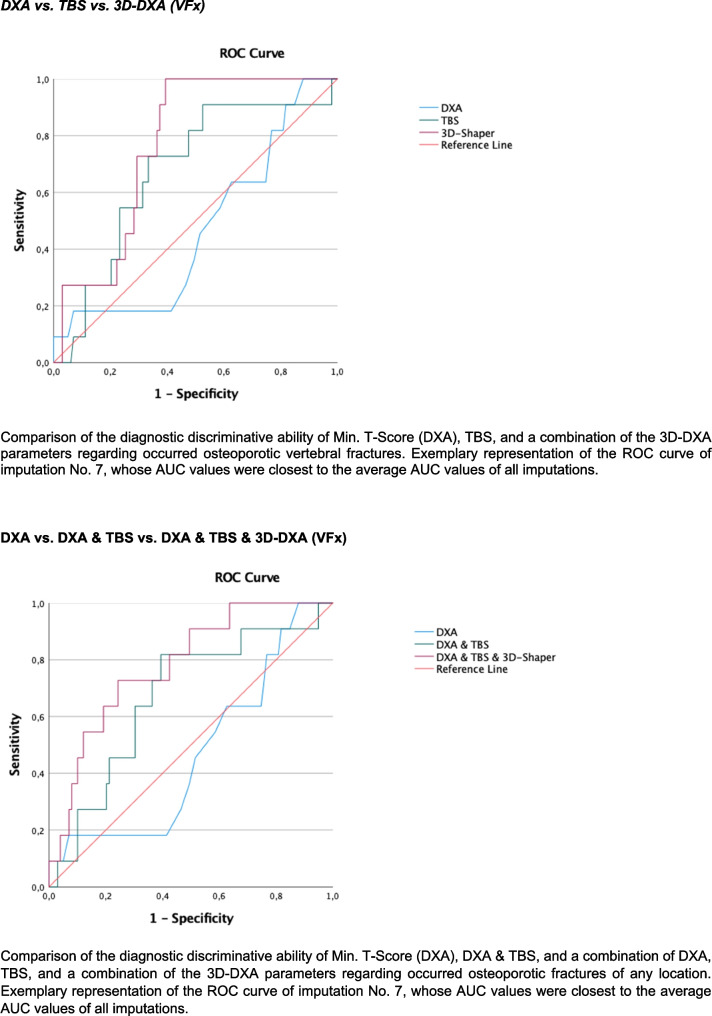
Discrimination performance of DXA, TBS, and 3D-DXA parameters for vertebral fractures

**Fig. 3:**
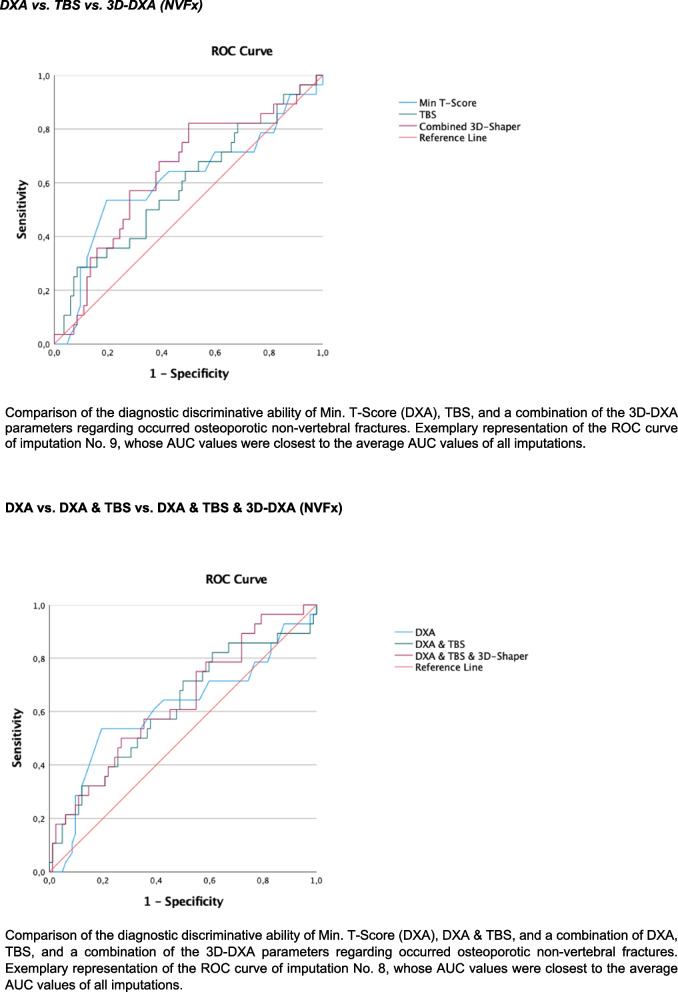
Discrimination performance of DXA, TBS, and 3D-DXA parameters for non-vertebral fractures

DXA-based T-Scores (MinTScore) reached AUC values of 0.61 (0.49; 0.73) for any prevalent fragility fractures (FFx), 0.47 (0.30; 0.64) for vertebral fractures (VFx), and 0.61 (0.48; 0.74) for non-vertebral fractures (NVFx), respectively (Figs. 1, 2, and 3). In comparison, the combination of 3D-DXA parameters for VFx showed AUC values of 0.77 (0.67; 0.87), 0.66 (0.54; 0.77) for any FFX, and 0.64 (0.52; 0.76) for NVFx. The results of tenfold cross-validation, as indicated by mean squared error (MSE) values, showed no improvement in model performance (see Supplementary Table 2) comparing DXA-based T-scores, TBS, 3D-DXA parameters, and their possible combinations.

## Discussion

Our analysis of a well-documented prospective cohort of 110 individuals with SLE revealed a prevalence of osteoporosis of 41%. Major osteoporotic fractures occurred in 29% of the patients. Multivariable linear regression analysis highlighted markers of disease activity such as high CRP, lupus nephritis class III and IV, the presence of U1-RNP antibodies and longer disease duration to be associated with low aBMD, while clinical remission was shown to be beneficial. For the composite measure of OP including fragility fractures, active LN was strongly associated with deteriorated bone health. Apart from these disease-specific factors, common risk factors such as higher age and low BMI as measures of functional disability showed to have a negative impact on bone. Structural 3D parameters of the femur showed similar discriminatory values for fracture detection compared to DXA and TBS.

OP in our cohort was shown to be a common comorbidity and complication in SLE affecting almost every second patient. The strongest factor determining an increased risk of OP in our study was lupus nephritis. LN is a severe manifestation of SLE, characterized by inflammation and damage to the kidneys. LN has previously been shown to be associated with an increased risk of OP and fracture in several studies [2]. A systematic review and meta-analysis of 23 studies with a total of 3,163 SLE patients found that the prevalence of osteoporosis was 17.7% in patients with LN compared to only 6.4% in patients without LN. Of note, the prevalence of vertebral fractures was 18.2% compared to 5.5%, respectively [1]. There are several mechanisms through which LN may contribute to bone loss. One is through the direct effects of inflammation and cytokine production, which can stimulate osteoclasts and inhibit osteoblasts, leading to net bone resorption. This is supported by studies showing elevated levels of pro-inflammatory cytokines in patients with LN, such as interleukin-6 and tumor necrosis factor-alpha, which have been associated with decreased bone mineral density. It is important to note that the severity of LN may also play a role in determining the risk of osteoporosis considering the effects of end-stage renal disease on bone. In our study, we found that patients with class III and IV nephritis were at higher risk for bone loss than other classes. This is consistent with previous research showing that more severe forms of LN are associated with higher levels of inflammation and more intensive use of GC therapy [26, 27], both of which can contribute to bone loss. The former is supported by our finding that high CRP levels negatively affect BMD. Systemic inflammation has long been shown to be a major driver of systemic bone loss [28–30]. This fact is very clearly confirmed in our cohort of patients with SLE.

Elevated Siglec-1 levels on monocytes, which in clinical practice are considered a marker of disease activity being a surrogate for Type-I interferon (IFN) activity in SLE [31], were associated with higher BMD values in our study. Little is known about the direct effects of IFN on bone cells and whether a stimulating effect can be observed. In fact, IFN type I signalling has been shown to prevent bone resorption by downregulating osteoclastogenesis in the context of systemic inflammation [32, 33]. Furthermore, positive effects of type I IFN signaling on the regulation of osteoblasts via Stat1 and Runx2 have been proposed [34]. However, the role of Siglec-1 on bone-metabolism is poorly understood, and when implicated with disease actitivity, the overall effects of disease activity and inflammation are likely to be negative on bone.

SLEDAI-2 K was paradoxically positively correlated with BMD, best explained by a skewed distribution (a majority of patients had SLEDAI-2 K values below 10). A potential selection bias could further contribute to this observation, as patients with recent onset of (highly active) disease may be more likely to undergo early osteoporosis screening. This finding is also contrasted by our observation that the presence of remission is beneficial to bone. A cumulative marker of disease activity, accounting for disease severity and duration over time, would likely offer more robust insights into the relationship between disease activity and bone health.

Another interesting finding refers to the presence of U1-RNP-antibodies, which were associated with worse bone health in terms of BMD. In patients with SLE, the presence of U1-RNP-antibodies was reported for 25–30% and is more reflective of musculoskeletal manifestations [35]. Our results may reflect a possible clinical subset of SLE patients at higher risk for systemic bone loss.

In our analysis, GCs did not have a distinct independent effect on bone. In the context of disease management, the known negative effects of GC on bone may be negligible if they are used with the aim of controlling disease activity at the lowest possible dose – an approach that is practiced in our centre. We have previously published that assessment of GCs in observational research needs to be contexualized with disease activity to reduce confounding [15]. Patients with LN had more often higher doses of GCs.

This study is the first to explore the potential role of DXA-derived 3D analysis of femoral bone structure in fracture discrimination among patients with SLE. While cross-validation showed that 3D-DXA parameters of cortical and trabecular bone did not significantly improve the discrimination of prevalent fragility fractures compared to DXA and TBS, there is a strong rationale for considering bone microstructure in fracture risk assessment. Due to the cross-sectional design, we analyzed only pre-existing fractures, often occurring years prior to the DXA assessment, in a relatively small patient cohort. Previous studies using HR-pQCT have demonstrated alterations in bone microarchitecture in SLE, including changes in trabecular structure [36] and reduced cortical thickness with higher cortical porosity in the distal radius [37]. Integrating 3D-Shaper technology with DXA may enhance fracture prediction. Future longitudinal studies assessing incident fractures could further clarify the predictive value of 3D femoral structural analysis alongside DXA and TBS.

A strength of our study is the comprehensive prospective collection of bone health data together with good characterization of the underlying disease, allowing for adjustment of known and potential new confounders. Thus, we were able to identify disease-specific factors affecting bone health in patients with SLE. Analysis of new techniques to evaluate bone health shed light on potential advances in fracture risk prediction. Being a monocentric cohort study of mostly white Caucasians may limit generalizability to other patient groups. A potential selection bias may have led to the fact that patients with more severe disease course already at high risk for osteoporosis were overrepresented in our tertiary university hospital [7]. Also, the cross-sectional design confines us to deduce associations rather than causality.

Future longitudinal analysis will allow us to better characterize the dynamics on bone health changes in patients with SLE and to pinpoint the role of the identified disease-specific factors.

To summarize, the identification of SLE-specific risk factors allows us to recognize patients at particular high risk for OP. This prompts us to suggest a thorough osteoporosis check-up in patients with high CRP, LN, or U1-RNP-antibodies.

## Supplementary Information

Supplementary File 1.

## Data Availability

The data underlying this study are not publicly available due to participant confidentiality agreements. Furthermore, the data are part of an ongoing cohort study, and public sharing is restricted to preserve data integrity for future planned analyses. Access to the data may be granted to qualified researchers upon reasonable request. For inquiries regarding data access, please contact the corresponding author, Edgar Wiebe (edgar.wiebe@charite.de, senior researcher at the Charité Universitätsmedizin Berlin).
