# Supplementary material for: Lupus nephritis and U1-RNP-antibodies are associated with low bone mineral density and osteoporosis in patients with systemic lupus erythematosus: baseline findings in a sub-cohort of patients with inflammatory rheumatic diseases
Source: Arthritis Res Ther. 2025 Jul 28;27:158. doi: 10.1186/s13075-025-03610-y (PMC12305897; doi:10.1186/s13075-025-03610-y)
Supplement: Supplementary file 1 [file 13075_2025_3610_MOESM1_ESM.docx]

**Supplementary material**

Table S1: Multivariable linear regression for 3D-DXA parameters

Table S2: Discriminatory performance of DXA, TBS and 3D-DXA parameters

Table S3: MSE of 10-fold cross-validation for prevalent FFx, VFx or NVFx comparing univariable und multivariable models with DXA-based T-Scores, TBS, 3D-DXA parameters.

Table S1 Multivariable linear regression

|  | **II) Integral vBMD** | | | **II) Trabecular vBMD** | | | **III) Cortical vBMD** | | | **IV) Cortical sBMD** | | | **V) Cortical thickness** | | |
| --- | --- | --- | --- | --- | --- | --- | --- | --- | --- | --- | --- | --- | --- | --- | --- |
|  | Reg. coeff. | (95% CI) | p-value | Reg. coeff. | (95% CI) | p-value | Reg. coeff. | (95% CI) | p-value | Reg. coeff. | (95% CI) | p-value | Reg. coeff. | (95% CI) | p-value |
| BMI | **2.5** | **(0.9;4.0)** | **0.002** | **2.3** | **(1.0;3.5)** | **0.001** | **3.9** | **(1.3;6.6)** | **0.004** | **1.6** | **(1.0;2.2)** | **0.001** | **2.5** | **(0.9;4.0)** | **0.002** |
| CRP | **-0.8** | **(-1.3;-0.3)** | **0.004** | **-0.6** | **(-1.1;-0.2)** | **0.004** | **-1.3** | **(-2.3;-0.4)** | **0.006** | **-0.3** | **(-0.5;-0.1)** | **0.007** | **-0.8** | **(-1.3;-0.3)** | **0.004** |
| SLEDAI-2K | **44.1** | **(20.8;68.2)** | **0.001** | 20.3 | (-0.5;41.0) | 0.055 | **61.8** | **(22.7;101.0)** | **0.002** | **20.3** | **(10.0;30.6)** | **0.001** | **44.1** | **(20.1;68.2)** | **0.001** |
| Anti-osteoporotic therapy | **-34.7** | **(−61.4;−8.0)** | **0.011** | **-23.3** | **(-44.5;-2.0)** | **0.032** | -39.3 | (-86.6;7.9) | 0.103 | **-12.9** | **(-24.1;-1.6)** | **0.026** | **-34.7** | **(−61.4;−7.9)** | **0.011** |
| Osteocalcin | **-1.2** | **(−1.9;−0.5)** | **0.001** | - | - | - | **-1.6** | **(-2.9 ;-0.4)** | **0.012** | **-0.4** | **(-0.7;-0.1)** | **0.004** | **-1.2** | **(−1.8;−0.5)** | **0.001** |
| Age (years) | - | - | - | **-0.6** | **(−1.1;-0.1)** | **0.030** | - | - | - | - | - | - | - | - | - |
| GC-duration (years) | - | - | - | -0.6 | (−1.3;0.1) | 0.088 | - | - | - | -0.3 | (-0.7;0.1) | 0.091 | - | - | - |
| Daily calcium intake | - | - | - | - | - | - | - | - | - | **8.2** | **(1.2;15.2)** | **0.021** | - | - | - |
| Elevated Siglec-1 levels | - | - | - | - | - | - | - | - | - | - | - | - | - | - | - |
| Female sex | - | - | - | - | - | - | - | - | - | - | - | - | - | - | - |
| Clinical remission | 14.8 | (-4.9;34.4) | 0.140 | 13.1 | (-2.7;29.0) | 0.104 | - | - | - | 7.4 | (-0.9;15.7) | 0.079 | 14.8 | (-4.9;34.4) | 0.140 |
| Vitamin D-deficiency | 23.6 | (−5.0;52.2) | 0.106 | - | - | - | - | - | - | 11.9 | (-1.0;24.8) | 0.070 | 23.6 | (−5.0;52.2) | 0.106 |
| Proteinuria | - | - | - | - | - | - | - | - | - | -0.01 | (-0.02;0.001) | 0.069 | - | - | - |
| Complementfactor 3 | - | - | - | −1.4 | (−4.2;1.4) | 0.322 | - | - | - | - | - | - | - | - | - |
| PPI | - | - | - | 5.8 | (0.4;-8.7) | 0.783 | - | - | - | - | - | - | - | - | - |
| HAQ | - | - | - | 8.3 | (−0.25;16.8) | 0.057 | - | - | - | - | - | - | - | - | - |
| Anti-SSA/Ro antibodies | - | - | - | 10.4 | (-2.9; -23.7) | 0.126 | - | - | - | - | - | - | - | - | - |
| Family history of OP | - | - | - | - | - | - | -25.9 | (-63.6;11.8) | 0.176 | - | - | - | - | - | - |

Table S2: Discriminatory performance of DXA, TBS and 3D-DXA parameters for any fragility fractures, and vertebral and non-vertebral fractures.

|  | **Fragility Fractures** | **Vertebral Fractures** | **Non-vertebral Fractures** |
| --- | --- | --- | --- |
|  | *AUC (95% CI)* | *AUC (95% CI)* | *AUC (95% CI)* |
|  |  |  |  |
| Min. T-Score (DXA) | 0.61 (0.49;0.73) | 0.47 (0.30;0.64) | 0.61 (0.48;0.74) |
| TBS | 0.62 (0.50;0.74) | 0.67 (0.51;0.83) | 0.59 (0.46;0.72) |
|  |  |  |  |
| Integral vBMD | 0.59 (0.47;0.71) | 0.55 (0.37;0.74) | 0.55 (0.43;0.68) |
| Trabecular vBMD | 0.60 (0.48;0.72) | 0.59 (0.41;0.77) | 0.56 (0.43;0.68) |
| Cortical vBMD | 0.55 (0.43;0.67) | 0.49 (0.31;0.68) | 0.52 (0.40;0.64) |
| Cortical sBMD | 0.55 (0.43;0.68) | 0.46 (0.27;0.66) | 0.53 (0.40;0.66) |
| Cortical thickness | 0.54 (0.42;0.66) | 0.51 (0.32;0.70) | 0.53 (0.40;0.66) |
| Combined 3D-DXA | 0.66 (0.54;0.77) | 0.77 (0.67;0.87) | 0.64 (0.52;0.76) |
|  |  |  |  |
| DXA & TBS | 0.65 (0.53;0.76) | 0.67 (0.51;0.83) | 0.61 (0.49 0.74) |
| DXA & TBS & 3D-DXA | 0.67 (0.56;0.78) | 0.78 (0.65;0.91) | 0.63 (0.51;0.75) |

Table S3: MSE of 10-fold cross-validation for the three endpoints prevalent FFx, VFx or NVFx comparing univariable und multivariable models with DXA-based T-Scores, TBS, 3D-DXA and the possible combinations of them. Shown are the pooled results of the 10 multiple imputed datasets.

|  | **Any FFx** | **VFx** | **NVFx** |
| --- | --- | --- | --- |
| DXA | 0.2918182 | 0.1000000 | 0.2545455 |
| TBS | 0.3136364 | 0.1000000 | 0.2590909 |
| 3D | 0.3027273 | 0.1054545 | 0.2627273 |
| DXA-TBS | 0.3100000 | 0.1000000 | 0.2645455 |
| DXA-3D | 0.3063636 | 0.1072727 | 0.2654545 |
| TBS-3D | 0.2763636 | 0.1100000 | 0.2645455 |
| DXA-TBS-3D | 0.2754545 | 0.1100000 | 0.2654545 |
